# Implication of comorbidity on the initiation of chemotherapy and survival outcomes in patients with locoregionally advanced nasopharyngeal carcinoma

**DOI:** 10.18632/oncotarget.8621

**Published:** 2016-04-06

**Authors:** Rui Guo, Yan-Ping Mao, Lei chen, Ling-Long Tang, Guan-Qun Zhou, Li-Zhi Liu, Li Tian, Mu-Sheng Zeng, Wei-Hua Jia, Jian-Yong Shao, Ai-Hua Lin, Jun Ma

**Affiliations:** ^1^ Department of Radiation Oncology, Sun Yat-sen University Cancer Center, State Key Laboratory of Oncology in Southern China, Collaborative Innovation Center of Cancer Medicine, Peoples Republic of China; ^2^ Imaging Diagnosis and Interventional Center, Sun Yat-sen University Cancer Center, State Key Laboratory of Oncology in Southern China, Collaborative Innovation Center of Cancer Medicine, Peoples Republic of China; ^3^ Sun Yat-sen University Cancer Center, State Key Laboratory of Oncology in Southern China, Collaborative Innovation Center of Cancer Medicine, Peoples Republic of China; ^4^ Department of Molecular Diagnostics, Sun Yat-sen University Cancer Center, State Key Laboratory of Oncology in Southern China, Collaborative Innovation Center of Cancer Medicine, Peoples Republic of China; ^5^ Department of Medical Statistics and Epidemiology, School of Public Health, Sun Yat-sen University, Guangzhou, Peoples Republic of China

**Keywords:** comorbidity, Initiation of chemotherapy, nasopharyngeal carcinoma, treatment outcome

## Abstract

**Background:**

To assess the impact of comorbidity on the initiation of chemotherapy and its ultimate treatment outcomes in patients with locoregionally advanced nasopharyngeal carcinoma (NPC).

**Methods:**

Data on 1316 patients with NPC treated between February 2003 and January 2007 was retrospectively reviewed. Comorbidity was assessed using the Adult Comorbidity Evaluation-27 (ACE-27) system. The association of various factors with chemotherapy was evaluated. And treatment outcomes of chemoradiotherapy regimes in patients with comorbidity were compared.

**Results:**

Comorbidity was present in 42.2% of patients; mild, moderate and severe comorbidity were observed in 33.6%, 8.1% and 0.5% of patients, respectively. Comorbidity (as indicated by ACE-27 score) was a negative prognostic factor for overall survival (OS) (hazard ratio HR=1.577; *P* < 0.001) and disease-free survival (DFS) (HR=1.509; P < 0.001). In stage III-IV NPC, T classification, N classification, age, sex and hemoglobin before treatment were significant predictors of the initiation of chemotherapy (P < 0.05). Additionally, in stage III-IV patients with comorbidity (ACE >0), 5-year OS for the concomitant chemoradiotherapy group (CCRT) was 74.5% vs. 56.9% in the radiotherapy (RT) only group (P = 0.008), the 5-year DFS rate was 64.0% in the CCRT group vs. 49.4% for RT only (P = 0.015).

**Conclusions:**

Comorbidity should be assessed during treatment strategy decision-making to improve survival in NPC. Concomitant chemoradiotherapy is feasible and effective in patients with comorbidity in locoregionally advanced stages.

## INTRODUCTION

Nasopharyngeal carcinoma (NPC) has a distinct epidemiology and geographic distribution, with the highest incidence of 20-50 cases per 100,000 males in Southern China [1]. Radiotherapy (RT) is the primary treatment modality for non-disseminated NPC due to its anatomical location and radiosensitivity [2]; however, the results with radiotherapy alone for locoregionally advanced NPC patients are usually unsatisfactory with 5-year overall survival (OS) rates of 67%-77% in stage III-IVB [3]. Various chemoradiotherapy (CRT) strategies have been explored, including concomitant chemoradiotherapy (CCRT), induction chemotherapy (IC) and adjuvant chemotherapy (AC)[4].

At present, standard clinical practice is to stage NPC according to the tumor-node-metastasis (TNM) staging system, which is based on the anatomic extent of the tumor and excluded patient-based prognostic factors. Comorbidity which is defined as the presence of medical ailments not caused by the primary tumor may add difficulty to treatment decisions for the use of chemotherapy [5]. In addition, as CRT strategies are widely used, it may exacerbate comorbidity, which may ultimately compromise survival or quality of life [6]. Therefore, The risks, decision making and potential benefit of CRT are necessary to assess for NPC patients with comorbidity before treatment.

Previous studies showed that patients with comorbidities received less, similar or more frequent uses of chemotherapy in different solid tumor [6]. However, little is known about outcome of CRT in patients with comorbidity, as comorbidities are generally not considered in the design of cancer data sets or assessed in observational research on the selection of CRT [7]. For NPC patients, no studies have investigated the impact of comorbidity on the selection of CRT and clinical outcome in NPC. We conducted this retrospective study to assess the impact of comorbidity on treatment selection and its ultimate effect on survival, in order to help optimize cancer care for patients with NPC undergoing CRT.

## RESULTS

The median follow-up was 75.3 months (range, 2.7-126.5 months). A total of 125/1316 (9.5%) patients developed local failure, 52/1316 (4.9%) developed regional failure, 19/1316 (1.4%) developed both local and regional failure, 233/1316 (17.7%) developed distant metastases, and 291/1316 (22.1%) patients died. The 5-year DFS, OS, DMFS and LRRFS rates were 71.7%, 79.5%, 82.5% and 87.9%, respectively.

### Differences of the patients with or without comorbidity

The overall distribution of stage I, II, III and IVA-B diseases was 8.7%, 25.4%, 36.9% and 29.0%, respectively. There was no association between disease stage and comorbidity, as indicated by ACE-27; however, the stage III-IV NPC patients with comorbidity (ACE-27 > 0) significantly received decreased chemotherapy compared with patients without comorbidity (67.6%vs.74.0%; P = 0.037). Furthermore, the significant associations were also observed between comorbidity and gender, age and type of radiotherapy (P < 0.05; Table 1).

**Table 1:** Characteristics of the 1316 patients with nasopharyngeal carcinoma

Patient characteristics	Without comorbidity (ACE-27=0)	With Comorbidity (ACE-27>0)	P-value
**No**	761	555	
**Age**			<0.001
< 45 years	444 (58.3)	239(43.1)	
≥ 45 years	317(41.7)	316(56.9)	
**Gender**			<0.001
Male	537 (70.6)	449 (80.6)	
Female	224 (29.4)	106 (19.1)	
**Histology**			0.660
WHO type I	4 (0.5)	2 (0.4)	
WHO type II/III	757(99.5)	553 (99.6)	
T classification*			0.744
T1	171 (22.5)	119 (21.4)	
T2	175 (23.0)	128 (23.1)	
T3	254 (33.4)	177 (31.9)	
T4	161 (21.2)	131 (23.6)	
N classification*			0.203
N0	211 (27.7)	177 (31.9)	
N1	344 (45.2)	235 (42.3)	
N2	147 (19.3)	92(16.6)	
N3	59 (7.8)	51 (9.2)	
Clinical stage*			0.339
I	63 (8.3)	52 (9.4)	
II	201 (26.4)	133 (24.0)	
III	289 (38.0)	197 (35.5)	
IVA-B	208 (27.3)	173 (31.2)	
**Radiotherapy**			0.002
2-DRT	418 (54.9)	352 (63.4)	
3-DRT or IMRT	343 (45.1)	203 (36.6)	
**Chemotherapy in stage III-IV**			0.037
RT only	129 (26.0)	120 (32.4)	
RT+chemotherapy	368 (74.0)	250 (67.6)	

Abbreviations: WHO, World Health Organization; 2-DRT, two-dimensional radiotherapy; 3-DCRT, three-dimensional conformal radiotherapy; IMRT, intensity-modulated radiotherapy; RT

* According to the 7th AJCC/UICC staging system.

### Prevalence, types and prognostic value of comorbidity

Of the 1316 patients, 555 (42.2%) had one or more comorbidity; 442 (33.6%) patients had ACE-27 scores of 1, 106 (8.1%) had scores of 2, and 7 (0.5%) had scores of 3. Gastrointestinal disease (24.9%), substance abuse (12.9%) and cardiovascular disease (5.4%) were most frequently observed (Table 2).

**Table 2:** Presence and severity of comorbidity in the study population of 1316 patients with NPC

Disease classification according to ACE-27	Grade 1: mild	Grade 2: moderate		Grade 3: severe
Overall ACE27 score	442 (33.6%)	106 (8.1%)		7 (0.5%)
Specific ACE27 categories				
Cardiovascular system	64 (4.9%)	7 (0.5%)	0 (0.0%)	
Respiratory system	25 (1.9%)	4 (0.3%)	0 (0.0%)	
Gastrointestinal system	317 (24.1%)	11 (0.8%)	0 (0.0%)	
Renal system	34 (2.6%)	0 (0.0%)	0 (0.0%)	
Endocrine system	21 (1.6%)	2 (0.2%)	0 (0.0%)	
Neurological system	6 (0.5%)	0(0.0%)	0 (0.0%)	
Psychiatric	1 (0.1%)	0 (0.0%)	0 (0.0%)	
Rheumatologic	2 (0.2%)	0 (0.0%)	0 (0.0%)	
Immunological system	0 (0.0%)	0 (0.0%)	0 (0.0%)	
Malignancy	4 (0.3%)	5 (0.4%)	5 (0.4%)	
Substance abuse	89 (6.8%)	80 (6.1%)	0 (0.0%)	
Body weight	1 (0.1%)	1 (0.1%)	0 (0.0%)	

Abbreviation: ACE-27, Adult Comorbidity Evaluation-27.

**Figure 1:**
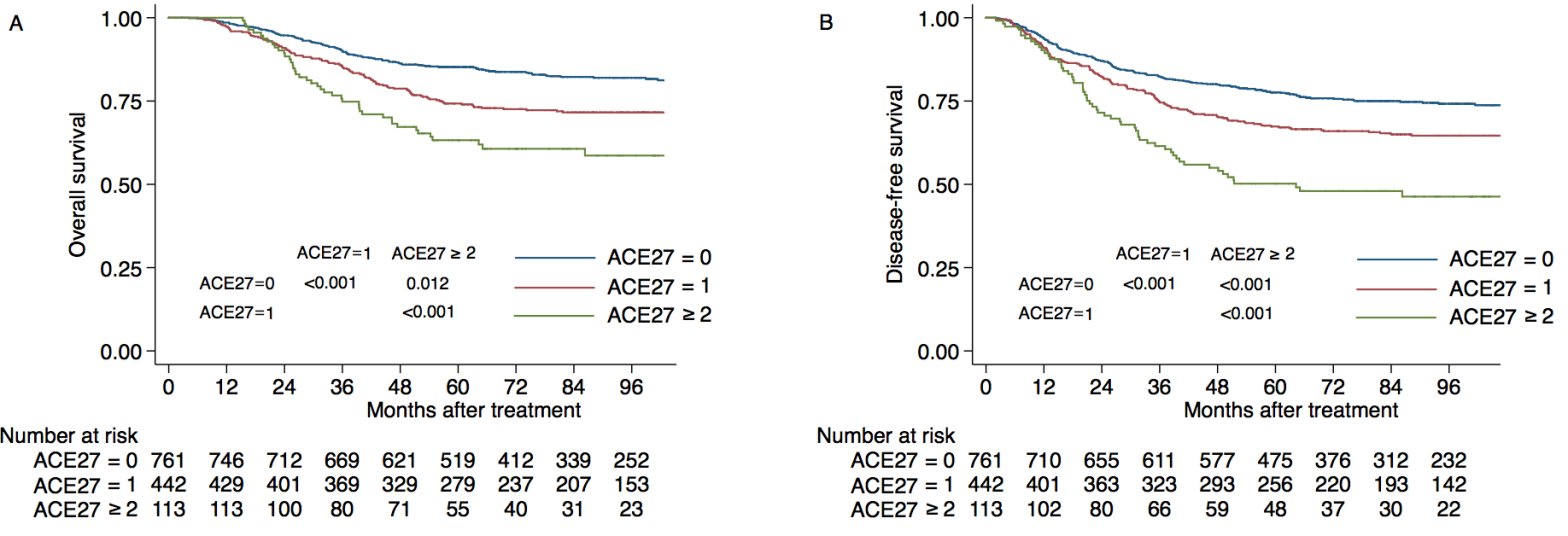
Kaplan-Meier estimates of survival for the 1316 study patients with nasopharyngeal cancer, according to ACE27 grade **A.** Overall survival, **B.** disease-free survival.

In univariate analysis, the 5-year OS rates of patients with ACE-27 grades of 0, 1 and ≥ 2 were 85.2%, 74.3% and 63.2% (P < 0.001; Figure 1A). The 5-year DFS rates of patients with ACE-27 grades of 0, 1 and ≥ 2 were 77.6%, 67.4% and 50.2% (P < 0.001; Figure 1B). In multivariate analysis, ACE-27 had significant independent prognostic value for OS (hazard ratio [HR] = 1.577; P < 0.001) and DFS (HR = 1.509; P < 0.001; Table 3).

**Table 3:** Multivariate analysis of the impact of all variables on survival

Endpoint	Variable	HR	HR (95% CI)	P-value‡
**Overall survival**	ACE-27	1.577	1.345-1.850	<0.001
	T classification*	1.548	1.374-1.744	<0.001
	N classification*	1.695	1.499-1.916	<0.001
	Age	1.376	1.088-1.740	0.008
**Disease-free survival**	ACE-27	1.509	1.314-1.734	<0.001
	T classification*	1.452	1.314-1.605	<0.001
	N classification*	1.527	1.373-1.698	<0.001

Abbreviation: ACE-27, Adult Comorbidity Evaluation-27; CI, confidence interval; HR, hazard ratio.

*According to the 7th AJCC/UICC staging system.

‡ Multivariate P values were calculated using an adjusted Cox proportional-hazards model. The following parameters were included in the Cox proportion hazard model by backward elimination: age, gender, World Health Organization (WHO) histological grade, T classification, N classification, radiotherapy (conformal vs. 3D and intensity modulated radiation therapy), use of chemotherapy (with vs. without) and ACE-27.

### Factors associated with initiation of CRT in advanced stage disease

For stage III-IV NPC patients, T classification, N classification, age, sex and hemoglobin before treatment and comorbidity (with vs. without) were significantly associated with the initiation of CRT in univariate analysis (P < 0.05; Table 4). And the T classification, N classification, age, sex and hemoglobin before treatment were significantly associated with the initiation of CRT in multivariable logistic regression (P < 0.05; Table 4). Furthermore, patients with a higher renal system burden and substance abuse were less likely to receive CRT (P = 0.068; P = 0.017; Table 4).

**Table 4:** Logistic regression analyses of factors associated with the uptake of chemotherapy by patients with stage III-IV nasopharyngeal carcinoma

Characteristic	No.	HR	95% CI for HR	Univariate *P*-value†	Multivariate *P*-value‡
**Age (y)**	867	0.977	0.961-0.993	<0.001	0.003
**Sex**				0.008	<0.001
Male	649	1	Reference		
Female	218	0.317	0.196-0.512		
**Histological type**				0.362	NS
WHO type I	4		Reference		
WHO type II-III	863	1.392	0.169-11.443		
Hematology					
Hemoglobin	867	0.968	0.955-0.981	<0.001	<0.001
Platelet	867	1.001	0.995-1.007	0.907	NS
White blood cell	867	0.860	0.590-1.254	0.110	NS
Neutrophil	867	1.077	0.671-1.729	0.076	NS
**Comorbidity present (Overall)**				0.037	0.089
Cardiovascular system	867	0.999	0.495-2.017	0.300	NS
Respiratory system	867	0.756	0.282-2.024	0.264	NS
Gastrointestinal system	867	0.890	0.625-1.266	0.212	NS
Renal system	867	0.372	0.128-1.077	0.077	0.068
Endocrine system	867	0.784	0.246-2.498	0.159	NS
Neurological system	867	0.899	0.112-7.220	0.580	NS
Malignancy	867	1.165	0.280-4.849	0.751	NS
Substance abuse	867	0.596	0.434-0.820	0.020	0.001
Staging					
**T classification§**					
T1	62	1.00	Reference		
T2	82	0.218	0.085-0.554	0.507	0.001
T3	431	0.219	0.095-0.502	0.109	<0.001
T4	292	0.462	0.297-0.721	0.001	0.001
**N classification§**					
N0	184	1.00	Reference		
N1	334	0.123	0.049-0.310	<0.001	<0.001
N2	239	0.206	0.085-0.499	0.049	<0.001
N3	110	0.363	0.163-0.808	0.038	0.013

Abbreviations: ACE-27, Adult Comorbidity Evaluation-27; CI, confidence interval; HR, hazard ratio; NS, not statistically significant; RT, radiotherapy; WHO, World Health Organization.

† Univariate *P* values were calculated using the binary logistic regession model.

‡ Multivariate *P* values were calculated using the binary logistic regession model.

§ According to the 7th AJCC/UICC staging system.

### Effect of comorbidity on CRT treatment outcomes

**Figure 2:**
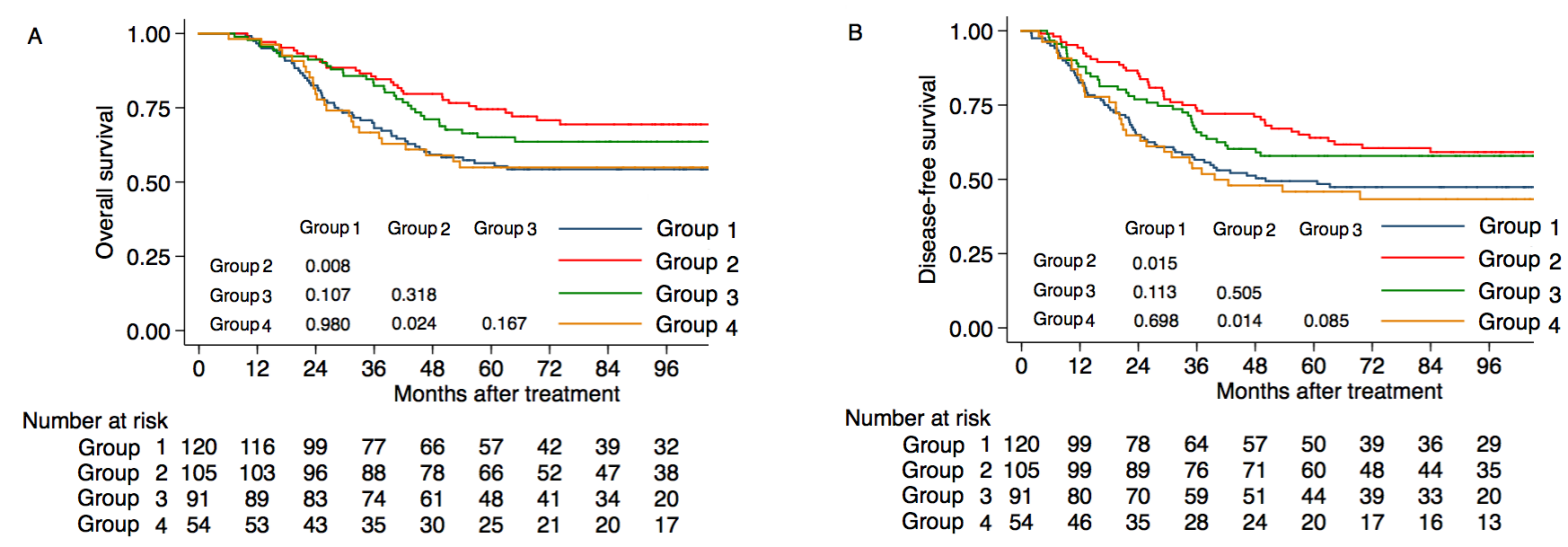
Kaplan-Meier overall survival curves for patients with Stage III-IV nasopharyngeal cancer according to the chemotherapy strategy A. Overall survival for patients with comorbidity (ACE scores > 0); B. disease-free survival for patients with comorbidity (ACE scores > 0). Group 1: radiotherapy (RT) only; Group 2: concomitant chemoradiotherapy (CCRT); Group 3: concomitant chemoradiotherapy plus induction chemotherapy/adjuvant chemotherapy (CCRT + IC/AC): Group 4: induction chemotherapy or adjuvant chemotherapy (IC/AC).

For stage III-IV patients with a comorbidity (ACE > 0), the 5-year OS rate in the CCRT group was 74.5% vs. 56.9% for RT only (P = 0.008), 65.0% for CCRT + AC/IC (P = 0.318) and 54.9% for IC/AC (P = 0.024; Figure 2A). The 5-year DFS rates was 64.0% in the CCRT group vs. 49.4% for RT only (P = 0.015), 57.9% for CCRT + AC/IC (P = 0.505) and 45.9% for IC/AC (P = 0.014; Figure 2B).

## DISCUSSION

Patients with comorbidities present considerable challenges to cancer management because they are often excluded from clinical trials. To our knowledge, this is a single-institutional study with the largest sample size to report the impact of comorbidity on the uptake of chemotherapy in NPC patients.

### The prevalence of comorbidity for NPC in south China

Comorbidities occur in 33%-65% of patients with head and neck cancer, with cardiovascular and pulmonary diseases most common [7–13]. In our research, 42.2% of patients with NPC had comorbidity; however, the most common comorbidities were gastrointestinal disease, which, according to the ACE-27, includes liver disease. This was not surprising as southern China has one of the highest incidences of chronic hepatitis B virus (HBV) infection, with hepatitis B surface antigen (HBsAg) positivity rates of 10-12% in the general population [14]. Therefore, chronic HBV infection may be an important gastrointestinal comorbidity in patients with NPC in southern China. An increased frequency and severity of comorbidity were noted with increasing age and in male patients. Piccirillo et al. also reported that increased age was associated with an increasing number and severity of comorbidities [15] with similar results obtained in other studies [13, 16, 17]. In this study, the second most frequent comorbidity was substance abuse, mainly in male patients.

### Factors associated with the initiation of chemotherapy in advanced stage NPC

Age, T classification, N classification, pretreatment hemoglobin were independent predictive factors for initiation of CRT; in agreement with previous studies demonstrating elderly patients tend to receive less intensive treatment [18]. However, The reduction in the use of CRT was only in patients with renal disease. Chemotherapy strategies for NPC include cisplatin, the use of which is limited by its severe acute and chronic nephro-, oto- and neuro-toxicity[19]. In everyday practice, the initiation of CRT is not primarily determined by comorbidity conditions, but renal disease.

### Implications of CRT in patients with comorbidity

Little is known about the toxicity and outcomes of CRT regimes in patients with s comorbidity, as these patients are often excluded from clinical trials. Our results indicate CCRT improved OS compared to RT only for patients with mild, moderate and severe comorbidity. Nevertheless, adding IC or AC to CCRT had no significant OS benefit. CCRT also improved OS compared to RT alone in patients with comorbidity (ACE > 0). Therefore, if patients with advanced NPC and comorbidity desire curative treatment, we consider CCRT to be the most appropriate approach; further studies are required to identify more intensive systemic approaches and novel agents, such as molecular-targeted agents, to improve the treatment outcomes for these patients. Moreover, patients with comorbidity should receive complete supportive care during treatment to ensure optimal outcome. ACE-27 requires no special technological expertise and can be applied by any healthcare professional. Comorbidity assessment could easily be included in research into prognostic factors and could represent a major confounder in clinical trials.

Since this was a retrospective sample, we had no control over the nature and quality of the medical records. Nevertheless, the records were detailed and provided accurate information on the patients’ clinical characteristics. More importantly, these results clearly demonstrate that comorbidity must be routinely considered with other significant prognostic factors in both clinical work and research. This study also justifies the need for prospective collection of comorbidity data in routine clinical practice.

## CONCLUSIONS

Comorbidity information should be incorporated into treatment strategy decision-making processes, to aid patient consultation and improve clinician decision-making. Concomitant chemoradiotherapy is feasible and effective in patients with comorbidity in locoregionally advanced stages.

## MATERIALS AND METHODS

### Patient selection

Between February 2003 and January 2007, 1403 newly-diagnosed patients with non-metastatic, histologically-proven NPC at Sun Yat-sen University Cancer Center (Guangzhou, People’s Republic of China) were retrospectively reviewed. Of these, 87 were excluded due to a lack of substantial data on comorbidity; the remaining 1316 cases were included. This retrospective analysis of the patient data was approved by the ethics committee of Sun Yat-sen University Cancer Center. Written consent was waived, while oral consent from the patients was obtained via telephone and documented by telephone recording.

All patients completed pretreatment evaluations, including complete medical history, physical examination, MRI of the nasopharynx and neck, hematology and biochemistry profiles, abdominal ultrasonography, chest radiography and whole body bone scan using single photon emission computed tomography (ECT); 139 (10.6%) patients also underwent positron emission tomography CT (PET-CT). All patients were restaged according to the 7th edition of the International Union against Cancer/American Joint Committee on Cancer (UICC/AJCC) system [20]. All MRI records were separately reviewed by two radiologists to minimize heterogeneity, disagreements were resolved by consensus.

### Comorbidity assessment

Comorbidity was evaluated by the Adult Comorbidity Evaluation-27 (ACE-27), a validated 27-item comorbidity index specifically developed for head and neck cancer [21–23]. The detailed information on the patients’ baseline medical condition and comorbidities (collected before diagnosis of primary NPC) were reviewed by one physician for ACE-27 scores. ACE-27 defines specific conditions using three grades (grade 0 = none; 1 = minimal; 2 = moderate; 3 = severe) according to organ system decompensation. The overall comorbidity score for each patient was based on the highest-ranked single ailmen. When two or more moderate ailments occurred in different organ systems, the overall comorbidity score was graded as severe.

### Treatment

Radiotherapy: All patients underwent definitive RT as reported previously [24–26]; 770/1316 (58.5%) received two-dimensional radiotherapy (2D-CRT) and 546/1,316 (41.5%) received three-dimensional conformal radiotherapy (3-DCRT) or intensity-modulated radiotherapy (IMRT).

Chemotherapy: The majority of the patients (618 of 867; 71.3%) with stage III or IV NPC (classified as T3-T4 and = or N2-N3 disease) received chemotherapy, including CCRT+/-IC/AC. Of these, 28.3% (245/867) patients received CCRT only, 30.8% (267/867) received CCRT+IC/AC, 12.2% (106/867) received RT+IC/AC. IC or AC consisted of cisplatin (80 mg/m2) with 5-fluorouracil (800 mg/m2/day over 120 h), or cisplatin (80 mg/m2) with taxanes (80 mg/m2) every 3 weeks for two or three cycles. CCRT consisted of cisplatin (80 or 100 mg/m2) on weeks 1, 4 and 7 of radiotherapy, or cisplatin (40 mg/m2) weekly.

### Follow-up and statistical analyses

The following end points (time to the first defining event) were assessed: overall survival (OS), disease-free survival (DFS), locoregional relapse-free survival (LRRFS) and distant metastasis-free survival (DMFS). Follow-up was calculated from first day of therapy to the day of death or last examination. Patients were followed-up every 3 months during the first 2 years, and every 6-12 months thereafter until death.

We used univariate analyses to examine the association of various factors with CRT. Then, multivariable logistic regression was used to calculate adjusted hazard ratio HRs. Actuarial rates were estimated by the Kaplan-Meier method, survival curves compared using the log-rank test. Multivariate analysis using a Cox proportional hazards model was used to test the different factors by backward elimination. Host factors (sex, age, ACE-27), tumor factors (histology, T and N classification), and treatment factors (RT technique and chemotherapy) were included as covariates in all analyses. Stata Statistical Package (STATA 12; StataCorp LP, College Station, Texas, USA) was used for all analysis. All tests were two-sided, P < 0.05 was considered significant.
